# Case Report: Special recoveries from major mental illness in childhood: a case series

**DOI:** 10.3389/frcha.2025.1377547

**Published:** 2025-05-22

**Authors:** Elena S. Monarch, Sean Foss

**Affiliations:** Lyme and PANS Treatment Center, Hingham, MA, United States

**Keywords:** PANS (pediatric acute-onset neuropsychiatric syndrome), PANDAS (pediatric autoimmune neuropsychiatric disorders associated with streptococcal infection), Lyme disease, treatment-resistance, root causes, children and adolescents, dysbiosis, recovery

## Abstract

Sudden-onset, unexplained, and treatment-resistant neuropsychiatric symptoms have been reported in numerous pediatric patients. Prior to the identification of neuroimmune conditions including PANS and PANDAS, children with these conditions were diagnosed with psychiatric and neurological conditions and offered conventional psychiatric and therapeutic care. As connections between the immune and nervous systems become clearer, alternate curative treatments have emerged. This case series presents three pediatric patients' treatment experiences with sudden-onset severe neuropsychiatric symptoms such as disabling anxiety, tics, eating restriction, or hallucinations, and their full recoveries following treatment of their underlying infections, inflammatory responses, and gastrointestinal dysbiosis. Treatments included antimicrobials, nutraceuticals, probiotics, and dietary changes. Converging lines of research highlight the importance of considering neuroinflammatory conditions in the differential diagnosis of children with treatment-resistant neuropsychiatric symptoms.

## Introduction

Certain infections and their inflammatory sequelae appear to trigger “psychiatric disorders” in some children and adolescents. The most well-characterized infection associated with psychiatric illness is streptococcal infection which has been linked with a constellation of symptoms known as Pediatric Autoimmune Neuropsychiatric Disorder Associated with Streptococcal Infections (PANDAS). Children with this condition experience sudden onset severe anxiety, often in the form of obsessive-compulsive disorder (OCD), and/or motor abnormalities such as tics or chorea. Along with unexplained extreme anxiety and/or characteristics of a movement disorder, children with PANDAS can exhibit seemingly senseless rages or even life-threatening behaviors such as jumping out of moving vehicles, physical violence against family members, or extreme restricted eating. At times, their symptoms can be mistaken for oppositional defiance or conventional psychiatric disorders. As such, prior to the identification and characterization of PANDAS by Dr. Sue Swedo et al. (1), children with this disorder were misdiagnosed with other conditions such as conduct disorder, bipolar disorder, Tourette's syndrome, or anorexia nervosa. Once accurately diagnosed and medically treated, many children with PANDAS can recover and resume their lives (2–6).

Since the identification of PANDAS in the late 1990's, numerous other infections have been linked to severe mental illnesses. In 2015, Sutterland et al. (7) published a meta-analysis of 50 studies dating back to 1995 reporting a significant association between parasitic infection with Toxoplasma gondii and mental health conditions, including OCD, bipolar disorder, and schizophrenia. Furthermore, the meta-analysis revealed a statistically significant association between the *level* of toxoplasma antibodies detected and severity of mental illness. Similar correlations between the onset of OCD and various infections have been reported for decades. For example, the common bacterial infection, Mycoplasma pneumonia, and viral markers such as herpes simplex virus type 1 antibodies have been associated with OCD [e.g., (8–10)]. Additionally, it is known that patients with persistent Lyme and tick-borne diseases can present with marked neurological and psychiatric symptoms [e.g., (11–14)]. In a study published in the American Journal of Psychiatry, one-third of psychiatric inpatients showed signs of past infection with the Lyme spirochete (15). Likewise, a nationwide Danish study published by Fallon et al. (16) showed that patients hospitalized with Lyme disease had a 28% higher rate of mental disorders and twice the likelihood of attempting suicide *after* infection compared to people without Lyme disease. Given the evidence that other infections aside from streptococcus may be risk factors for a host of neuropsychiatric symptoms, Dr. Swedo et al., put forth a second and more inclusive clinical diagnosis termed Pediatric Acute-Onset Neuropsychiatric Syndrome [PANS; (17)]. This broader diagnostic syndrome applies to patients with sudden-onset neuropsychiatric symptoms associated with various known or even unknown etiologies including single or multiple infections, immune dysfunction, environmental irritants, or other sources of neuroinflammation without presumption of pathogenic mechanisms.

The role of various infections in triggering neuropsychiatric symptoms and their theorized mechanisms of action have been summarized elsewhere [e.g., (18)]. With increasing clarity of an association between OCD and immune dysregulation, several researchers have recommended subset-profiling OCD patients with known infectious triggers, including Lyme disease, and/or other autoimmune abnormalities (19, 20). Most recently, SARS-CoV-2 has been identified as a putative trigger of neuropsychiatric symptoms in children with authors asserting “SARS-CoV-2 needs to be acknowledged in the differential diagnosis of PANS” (21, 22). Nonetheless, when young patients experience sudden onset neuropsychiatric symptoms, it is not standard of care in psychology, psychiatry, or pediatric practices to evaluate infections, autoimmune or inflammatory markers, or blood brain barrier integrity.

Skepticism about the value of advancing a PANS/PANDAS diagnosis has been questioned with some researchers stating, “The evidence for use of antimicrobial and immune-based therapies in youth satisfying criteria for PANDAS/PANS is of low quality” and recommend continued preferential treatment with psychiatric medications for these children (23, 24). “Youth satisfying criteria for PANDAS/PANS should receive treatment with psychotropic medications and can be expected to show improvement rates similar to youth with OCD not satisfying PANDAS/PANS criteria” (25). Others are less enthusiastic about the universal benefits of psychotropic medications for youth suffering from PANDAS (26, 27) and instead advise a “start low and go slow” medication approach to avoid or foresee potential adverse reactions to psychotropics (3). With unclear or insufficient relief of neuropsychiatric symptoms from psychotropic medications, healthcare providers are in need of other treatment modalities. Hence the implementation of immunotherapies and alternative treatments, though these approaches have yet to establish convincing or comprehensive curative effectiveness. The lack of consistency in treatment effects could be due, in part, to the heterogeneity of the PANS patient population. Which irritants or infection(s) may have triggered an individual child to suffer with neuroinflammation? It is difficult, if not impossible, to appreciate all the mediating factors in a child whose neuroinflammatory autoimmune disorder has led them to obsessively lick their hands, fear toilets, drop out of school, or starve themselves due to contamination fears.

While the link between infections and neuropsychiatric conditions continues to be investigated and providers attempt to treat neuroimmune conditions with available modalities, compelling case study recoveries have been published [e.g., (6, 11, 28)]. The purpose of the current paper is to present three young patients’ complete recoveries from severe neuropsychiatric conditions following treatment of their tick-borne diseases and inflammation.

Considering that Lyme disease is the fasting growing infectious disease in the United States, exploring whether it triggers PANS is arguably of critical importance for the prevention of long-term neurocognitive deficits and psychopathology in our youth (16, 29, 46). In addition to the rising incidence of Lyme disease, adequate testing and diagnosis of this infection is known to be problematic. According to the Center for Disease Control (CDC), untreated Lyme disease symptoms can include neurological symptoms such as “problems with short-term memory”, “tingling, nerve pain”, “dizziness”, “headaches” and/or “inflammation of the brain and spinal cord”. Children, such as those described in this case series experienced some of those symptoms along with disabling neuropsychiatric symptoms that can be misdiagnosed as major mental illnesses rather than PANS or neuroinflammation.

## Methods

The authors requested consent from three patient families treated at the Lyme and PANS Treatment Center (LPTC). Outside of consenting, no research intervention or participation was requested of the patients in this retrospective chart review study. All three identified patients were minors at the time of their care at LPTC; one is now a young adult. Verbal and written informed consent were obtained from all three sets of parents. The two older patients also signed consent forms. Parents of the youngest patient, “Mia”, obtained her verbal assent. Given her very young age, it was their preference to confer with her. Due to the retrospective, anonymous, and non-interventional nature of this case series, the study was not eligible for institutional review by a board.

The authors read and summarized all available laboratory tests and clinical notes from the patients' paper charts and electronic health records, including lab results that patients may have completed elsewhere prior to or during their care at the LPTC.

## Results

### Case study #1

“Mia” initially presented at age 3 years and 6 months with sudden onset disabling obsessions, compulsions, and sensory sensitivities. Her mother reported it seemed as if suddenly “an alien took over her body”. Mia's new behaviors included licking her hands, tasting non-food items, hyper-focus on “fixing” her hair, needing to be close to her mother, and spending hours in the bathroom toileting. In addition to her OCD symptoms, Mia exhibited emotional and behavioral dysregulation described as “volatile mood”, anhedonia, and “picky eating”. In terms of physiological symptoms, Mia suffered with insomnia, physical restlessness, low-grade fevers, fatigue, increased sensory defensiveness, and inattentiveness in the form of “zoning out”.

#### Background

Mia lives with her parents and sibling in New England. One parent is a healthcare practitioner and was able to report comprehensive details about symptom onset. Mia's family medical history is positive for a mother with celiac and tick-borne diseases, and a maternal grandparent with Hashimoto's disease.

Mia's medical background included an uncomplicated pre-natal and perinatal history. At age 2, she experienced a two-week episode of unexplained limping. At 2 years and 6 months she had recurrent urinary tract infections for which she was treated with multiple courses of antibiotics. At 3 years and 1 month, she developed a kidney infection, was hospitalized for two nights, treated with intravenous fluids and antibiotics, and prescribed 10 additional days of oral antibiotics. During this episode Mia was tested for streptococcal infection with negative findings. Immediately following discharge from this hospitalization, Mia developed a respiratory illness (thought to be viral) followed by her first, albeit mild, psychiatric symptoms including anhedonia, picky eating, and moodiness. These transient symptoms were ascribed to her physical illness and adjustment to the birth of her younger sibling. Outside of these occurrences, Mia's developmental history was unremarkable, though her mother described Mia as having a rigid and headstrong personality.

At age 3 years and 6 months, Mia had another urinary tract infection and concomitant respiratory illness. This time Mia exhibited sudden onset severe and disabling neuropsychiatric symptoms to the point where her mother described her as unrecognizable. A visit to the pediatrician's office was inconclusive as lab work including throat culture, strep titers (ASO and Anti DNase B) and Lyme enzyme-linked immunosorbent assay (ELISA) were negative. In spite of these negative findings, Mia's mother suspected PANS and requested parenting support from a Psychologist at the LPTC.

#### Evaluation and treatment

At intake, Mia's severe OCD interfered with her ability to function, and the family was housebound. Parent report included “compulsively tasting odd non-food items”, habitually licking her hands, insisting on wearing a hat all day and night, nail biting, and an extensive toileting routine with repetitive wiping. Mia's mother said her daughter suddenly became “very anxious”, “extremely sensory defensive”, “rigid” in her routines and demands, and began “perseverating on scary topics”. Given that her pediatrician ruled out PANDAS and bacterial infections, Mia's mother sought psychoeducation and parenting support from the LPTC. While the psychologist visits were beneficial for the mother, Mia remained neuro-psychiatrically impaired with disabling OCD, and the family remained largely housebound. After four months in that state, the mother (a health care professional) enacted a “strict” anti-inflammatory diet along with supplements (e.g., magnesium) on her own. After four months of these interventions, Mia's symptoms slowly improved. After seven months of these interventions, Mia was able to return to preschool. She was reported to be “very happy” and “well-adjusted”. She remained well for two additional months.

At age 4 years and 8 months, following a viral illness, Mia experienced a second sudden onset severe neuropsychiatric episode. On this occasion, the parents sought medical evaluation and treatment at the LPTC. They explained that Mia had become extremely anxious again, behaviorally regressed, restricted her eating, and felt compelled to constantly “fix” her dolls' hair styles to the point where she could not focus on anything else. As part of the initial evaluation, Mia was tested for streptococcal infection; the culture was positive. Mia's mother also completed an empirically validated Lyme disease questionnaire (30) and her score of 51 placed Mia in the “high probability of a tick-borne disorder” range. Nonetheless, given her positive strep test and symptomatology, Mia was diagnosed with PANDAS, which was treated with two weeks of azithromycin, and four months of the following: probiotics, fish oil (Ultimate Omega by Nordic Naturals 1,280 mg daily), multivitamin (Pure Encapsulations Junior one capsule daily), and curcumin (Pure Encapsulations 250 mg daily). With these treatments in place, improved neuropsychiatric symptoms were evident after one week. One month into treatment Mia's OCD was still present but waning (e.g., changing her socks frequently, needing to “constantly adjust her headband”, and repeated wiping after urination). Two months later, however, her symptoms worsened again with a similar neuropsychiatric presentation. This time testing for streptococcal infection was negative. Due to the parents' exasperation and the child's degree of disability they requested more comprehensive testing, which included other infections, irritants, and markers of immune dysregulation. Test results were unremarkable except for Borrelia burgdorferi (see Table 1 for list of tests). Mia was diagnosed with PANS and Lyme disease and treated with a similar protocol as above including antibiotics, probiotics, fish oil, multivitamins, curcumin, and magnesium. Her neuropsychiatric symptoms began to resolve after three weeks and this time she remained well for two years. During the period of wellness, at age 6, Mia was seen by a psychologist at the LPTC for a “giftedness” evaluation per the parent's request. Mia's cognitive functioning was above average and she was free of OCD and other neuropsychiatric symptoms.

**Table 1:** Mia relevant test results.

Test panels	Laboratory	Interpretation
Food allergens and sensitivity serology	Vibrant Wellness	Numerous high and moderate IgG and IgA sensitivities
Micronutrient deficiency	Spectracell	B12 and choline deficiencies
Pathogen serology	Quest Diagnostics Lab Galaxy Labs Igenex Arminlabs	Positive Borrelia burgdorferi, Babesia microti, and Bartonella henselae and quintana, First Streptococcal culture positive and second negative
Organic acids test and fungal antibodies	MOSAIC LABS Vibrant Wellness	Candida and bacterial dysbiosis, elevated oxalates
Basic blood work	Quest Diagnostics Labs Labcorp	Elevated TGF B-1, Leukopenia and Neutropenia
Mycotoxin	Vibrant Wellness Realtime Labs	High Patulin, Ochratoxin A and M1; moderate diacetoxyscirpenol
Heavy metals	Vibrant Wellness	No elevated Heavy Metals
Parasite stool histology	Parawellness	Endolimax nana- few to moderate amount

At age 7, Mia returned to LPTC a third time with severe sudden onset OCD. On this final occurrence, Mia was unable to touch things due to contamination fears. She held her hands in the air. As a result, she could not feed herself. On this re-occurrence, Mia suffered with intense anxiety and caution about germs, unrelenting perfectionism, physical restlessness, and insomnia. She began obsessively confessing small mistakes she had made in the past. Her mother described Mia as “a different person” than who she had been for the previous two years. Comprehensive testing was ordered. Lab work was largely negative including absence of streptococcal infection. However, Mia tested positive for (1) Babesia Microti, a tick-borne co-infection (blood test) (2) Ochratoxin A, Aflatoxin M1, Patulin mycotoxins (urine test), (3) three fungi including candida (stool test) and (4) two parasites (stool). Mia's treatments were introduced gradually and included probiotics, cod liver oil, curcumin, and alternating herbal antifungal and antiparasitic tinctures (e.g., Fungisode by Genestra/Seroyal Sudbury, MA USA; Cumanda by Nutramedix; Crypto-Plus manufactured by Researched Nutritionals, Los Olivos, CA, USA; Biocidin by Biocidin Botanicals 18 Hangar Way, Watsonville CA 95076 USA; BB1and BAB3 by Beyond Balance, Inc. Rohnert Park, CA USA). Mia also participated in a brief course of telehealth cognitive behavioral therapy (CBT). It was noted that Mia showed minimal improvement during CBT. The first signs of progress occurred six weeks after medical treatments with full recovery following an additional four weeks later. From that point until present day, her parents describe Mia as “100% recovered” and better than her premorbid status in that her personality is less rigid and more agreeable. Her most recent Lyme disease and Babesia microti tests were negative. Mia continues to take a multivitamin/multimineral, cod liver oil, and other routine supplements as needed (e.g., probiotics, vitamins D, C, and zinc). In addition to being free of neuropsychiatric symptoms for over three years, today Mia is an especially high functioning 11-year-old who obtains excellent grades, performs on stage, and enjoys extracurricular activities. Since her recovery, Mia had a recurrence of streptococcal infection without any neuropsychiatric impact.

### Case study #2

“Shelly” was referred for treatment at age 12 by her Occupational Therapist (OT). The OT contacted the LPTC stating that her formerly healthy and vibrant private OT client experienced a profound and rapid regression in physical, cognitive, and emotional health over the previous four months. At that time, Shelly was in the process of being discharged from her second lengthy hospitalization where she was diagnosed with “psychosomatic” and functional neurological disorders (FND), and chronic regional pain syndrome (CRPS).

#### Background

Shelly was born prematurely at 32 weeks and was treated in the NICU for 4 weeks. She was diagnosed with a milk allergy during infancy. Despite developmental delays she advanced through school very well until third grade when she experienced “abrupt anxiety” and new hoarding behaviors. Later that year she was diagnosed with an atypical seizure disorder, mononucleosis, and eventually Asperger's disorder for which she received speech and occupational therapies. With supports in place and anti-seizure medication, Shelly successfully completed public elementary school, but transitioned to homeschooling at age 12 during a period of illness. Shelly lives with her parents and siblings in New England. Two siblings have had Lyme disease.

#### Evaluation and treatment

Shelly's mother requested an in-home evaluation due to her daughter's inability to travel. Behavioral observations at intake included the following: “confined to a wheelchair”, “screeching, moaning, writhing”, “fearful and crying”, and “unable to speak but can answer some questions with nods of her head”. Shelly had limited motor control and was unable to feed herself. She was observed drinking an ice cream shake through a straw as her mother held the cup. Her entire diet was restricted to two foods. She was unable to speak but retained two words using sign language which she expressed during the visit: “sad” and “hurt”. Shelly's skin was pale and she was sweating profusely prompting her mother to dry her face with a towel. Although Shelly was very thin, her belly was prominent, distended, and the interview revealed that she was constipated.

Shelly's mother explained that her daughter experienced an abrupt uptick in anxiety five months earlier during an episode in which Shelly complained of sharp stomach and leg pains resulting in an emergency room visit and lengthy hospitalization. Outside of her intense pain complaints and heightened anxiety, at the time of admission Shelly was able to communicate, ambulate, and function normally most of the time. During the hospitalization, Shelly's mental and physical health declined, with escalating separation anxiety, intrusive thoughts, agitation, headaches, nausea, food restriction, and continued complaints of stomach and leg pain. Hospital records indicate eighteen medications were administered to no avail, including Celexa, Trileptal, and Lamictal. According to her mother, Shelly's regression during the hospital stay led to her losing language and motor skills, and the ability to care for herself. After two months in the hospital, when Shelly's pain was more tolerable, she was discharged from the hospital, and she convalesced at home for two weeks with her psychotropic medications. However, Shelly's symptoms did not improve at home and during another bout of severe stomach pain, she returned to the emergency room, and was admitted for two more months.

During her second hospital stay, the psychiatry team and other clinicians initiated a plan in which Shelly was to “earn” her time with her mother. It was their impression that Shelly's anxiety, agitation, and pain complaints were psychogenic and behaviorally reinforced. Behavioral treatment plans were put in place to lengthen the times of separation between Shelly and her mother. Shelly's mother reported this experience as “traumatic” for herself and daughter.

Shelly's second inpatient discharge took place when it was determined that she was not demonstrating progress in that setting. Discharge plans included psychotropic and continuation of anti-seizure medications, along with in-home applied behavioral analysis therapy which the family declined due to the pandemic.

Medical records from the two hospitalizations list chief complaints as “behavioral outbursts” and “agitation”. Records describe her cerebral spinal fluid as “normal”. MRIs of Shelly's spine and brain were described as “unremarkable”. A chart note states Lyme Elisa and streptococcal tests were negative.

After the in-home initial evaluation and review of the patient's hospitalization notes, lab work was ordered (see Table 2). The clinical interview revealed that two of Shelly's siblings had had tick bites, and Shelly had been exposed to ticks in her backyard with one possible attachment on her chest in the summer between second and third grade. Therefore, a tick-borne disease panel was included in her lab orders. While awaiting test results, Shelly's diet was adjusted. The ice cream shake meals were gradually replaced with dairy-free protein smoothies made with natural ingredients. Additionally, soft foods such as avocados and mashed potatoes were introduced. She also began drinking water with powdered magnesium citrate 200 mg daily. After two weeks, Shelly's pain symptoms lessened, and she started to speak single words again.

**Table 2:** Shelly relevant test results.

Test panels	Laboratory	Interpretation
Food allergens and sensitivity serology	Precision Point Diagnostics	Numerous high and moderate IgE allergies and IgG and C3D sensitivities
Organic acids test	MOSAIC LABS	Candida and bacterial dysbiosis
Basic blood work	Vibrant Wellness	Microcytic anemia
Parasite stool histology	Parawellness	Presence of Blastocystis hominis and Giardia intestinalis
MARCONS nasal culture	Microbiology DX	High Multiple Antibiotic Resistant Coagulase Negative Staphylococci infection
Pathogen serology	Quest Diagnostics Labs Medical Diagnostics Laboratory Mosaic Labs	Positive Borrelia burgdorferi HHV-6, Bartonella garinii, cytomegalovirus, EBV, Chlamydia pneumonia
Basic blood work	Quest Diagnostics Labs Labcorp	Elevated TGF B-1, Lymphocytosis and Neutropenia
Micronutrient deficiency	Vibrant Wellness Spectracell	Deficient carnitine, chromium, cysteine, Cu, Vitamin B1, B3 A and D
Hormones	Vibrant Wellness	Elevated estrogen and cortisol

Lab results were largely negative, however, the tick-borne disease panel revealed (IgM) positive Lyme disease via both Quest (using CDC surveillance criteria) and Medical Diagnostic Laboratories. Shelly also tested positive for small intestinal bacterial overgrowth (SIBO). Over the course of twelve months, Shelly received multidisciplinary care for Lyme disease and gut dysbiosis (SIBO and constipation). Given Shelly's history of severe stomach pain, she was not offered antibiotic medications. Instead, treatments included herbal antimicrobials (Mimosa Pudica 900 mg by Cellcore Biosciences Meridian, ID 83642), probiotics (Saccharomyces boulardii 10 billion CFU Pure Encapsulations, Sudbury, MA 01776), digestive enzymes (Designs for Health Palm Coast, FL 32164), magnesium citrate 200 mg for constipation, Omega 3 Fatty acids 600 mg (Nordic Naturals, Watsonville, CA 95076), monolaurin 3,000 mg (Med-Chem Labs Inc., Goodyear, AZ 85338), resveratrol (500 mg by Nutra BioGenesis, Salt Lake City, UT 84101), and nutrient supplementation (Ultra Clear by Metagenics, Aliso Viejo, CA 92656). Aside from her anti-seizure medication, Shelly's psychotropic medications were slowly tapered and eventually discontinued as none appeared to have benefited her symptoms. In the second and third months of treatment Shelly regained some skills and was able to feed herself again. At the start of the fourth month, herbal antibacterial and antifungal tinctures (Samento and Cumanda by Nutramedix) and modified citrus pectin (Allery Research Group) were gradually added to her protocol. By the end of six months Shelly experienced 75% recovery, speaking in sentences, toileting by herself, ambulating, and eventually holding a pencil and doing schoolwork. After eight months, Shelly returned to school part-time. One year into treatment, yoga therapy was added to her interventions for the purposes of learning self-calming techniques and preventing constipation. At the same time, Shelly began horseback riding as a leisure activity. Two months later, Shelly's mother reported that her daughter not only regained her physical and mental health but improved beyond her premorbid status in some areas. For example, Shelly's writing and drawing skills far exceeded prior achievements. Finally, Shelly's seizure disorder remitted, and her neurologist tapered the long-term anti-seizure medication without incident.

One year after her complete recovery, Shelly started psychotherapy at the LPTC to address post-traumatic stress related to her hospitalizations. Currently, at age 15, Shelly experiences mild vocal tics and signs of anxiety in the fall season but otherwise has remained healthy for over three years, functions well, and attends high school.

### Case study #3

“Tyler” presented at 13 years of age with recent diagnoses of Sydenham's chorea and PANS from a neurologist and infectious disease doctor, respectively. His parents explained that four months earlier, over the previous summer, their son had a “mysterious illness” with fever, fatigue, and anxiety. Several weeks after his mysterious illness, Tyler was prescribed Zoloft by a clinician to treat his anxiety. During titration Tyler developed more severe neuropsychiatric symptoms, including obsessions, fearfulness, intrusive thoughts, hallucinations, and paranoia, prompting their visits with a neurologist and infectious disease doctor. Six weeks after initiating treatments from those providers, Tyler did not show any signs of progress and the parents requested an intake at the LPTC.

#### Background

Tyler's pre and postnatal histories were described as normal. His early milestones were achieved on time. At 22 months of age Tyler inexplicably began losing his verbal language achievement and exhibited new sensory processing difficulties. He was subsequently diagnosed with a pervasive development disorder (“mild Asperger's Disorder”) and later diagnosed with an anxiety disorder. With occasional school accommodations and parental support, Tyler successfully advanced through elementary school. In the home setting, Tyler's parents described him as an easy, cooperative child until the summer between 6th and 7th grade when he “didn't look healthy”, “had problems with eating”, complained of gastrointestinal distress, aches and pains. Tyler began severely restricting his food intake and exhibited signs of anxiety. Tyler's parents sought treatment for their son which included psychotherapy by a therapist and 25 mg QD of Zoloft daily by a psychiatric nurse practitioner. One month later, in early September, the dose was increased to 50 mg daily. Three weeks later, in late September, Tyler decompensated further with his parents stating “he became psychotic” and developed motor tics with increasing intensity and frequency. In late September, a neurologist diagnosed Tyler with Sydenham's chorea, and an infectious disease doctor diagnosed him with PANS. A cardiac evaluation was completed to rule out rheumatic fever. Tyler was prescribed a host of medications by the aforementioned doctors including prednisone, guanfacine (2 mg QD), klonopin (0.5 mg BID), hydroxyzine (50 mg, PRN), and Augmentin 250 mg QD (due to a suspected strep infection). While Tyler continued his medications including 50 mg of Zoloft QD his symptoms worsened further prompting his parents to schedule an evaluation at the LPTC in November.

#### Evaluation and treatment

Upon arrival for his evaluation, Tyler's eye contact was poor and he made facial expressions of terror. He had exaggerated startle responses to minor sounds in the office, but also startled for no apparent reason. Tyler stood throughout this appointment (and the next 18 appointments). At intake, Tyler was too disabled to communicate or withstand a physical evaluation, hence his providers relied heavily on parent reports. They explained that Tyler could not be near his father in or outside of the office as he thought his father was “out to kill him”. Observationally, clinicians surmised that Tyler may have been hallucinating; this was confirmed six months later when Tyler was able to self-report. In addition to the symptoms above, Tyler developed a fear of bathrooms/showering, was no longer able to go to school, and his food restriction worsened. Despite Tyler's premorbid cooperative and polite demeanor, at the time of his intake his parents reported their son has intense “rages”. He was nearly hospitalized several times due to concerns about his safety. [Months later, Tyler was able to explain that he suffered with persistent intrusive thoughts, threatening auditory hallucinations, intense phobia of his father, and inability to sleep due to fear of being killed while asleep.] At intake, when asked to provide a rating of their son's wellness, both of his parents said “zero percent well”.

Based on clinical interviews, available medical records, and behavioral observations, LPTC clinicians concurred with Tyler's previous PANS diagnosis and initiated treatment. While awaiting lab results, Tyler was advised to increase hydration, take ibuprofen 4–10 mg/kg, Omnicef (300 mg BID), omega-3 fatty acids 900 mg BID, and slowly taper Zoloft. Four weeks later, at follow-up, his parents reported 30% improvement in symptoms, but continued to describe their son as extremely impaired, unable to sleep or shower, terrified of his father, and unable to attend school.

Lab results indicated that Tyler's streptococcal throat culture and bloodwork titers were negative. Elevated antibodies were detected for multiple tick-borne diseases*.* Considering the facts that Tyler' favorite pastime involved being in the woods collecting frogs, and he had a known history of tick bites from a Lyme endemic northeast region, the presence of tick-borne diseases was plausible. Other lab results included elevated anti-dopamine receptors (Cunningham panel, Moleculara Labs), elevated antibodies for viruses, and high IgG reactivity to wheat, gluten, cow's milk, and casein (see Table 3 for list of tests).

**Table 3:** Tyler relevant test results.

Test panels	Lab	Interpretation
Neurotransmitters	Sanesco International	Low Serotonin, GABA, Dopamine, Epinephrine, Glutamate, and DHEA-s; high Cortisol
Basic blood work	Quest Diagnostics Labs Vibrant Wellness	Elevated MPV and phosphate, Leukopenia, CD 57 low
Intestinal biome stool test	Diagnostic Solutions Lab	Intestinal dysbiosis
Cunningham panel	Moleculara Labs	Elevated anti-neuronal antibodies
Food allergens and sensitivity serology	KBMO Diagnostics	Numerous high and moderate IgG and C3D sensitivities
Pathogen serology	Quest Diagnostics Labs Igenex	Positive Borrelia burgdorferi, Francisella tularensis, cytomegalovirus, mycoplasma pneumoniae, coxsackie B, EBV, HSV1, HHV6

Due to Tyler's level of impairment, his parents and doctors opted to initiate high dose Intravenous immune globulin (IVIG). Other treatments included cefdinir (300 mg q12h), and additional supplements including vitamin D3 (2,000 IU QD, BCM-95), Turmeric Extract (500 mg QD), Saccharomyces boulardii (10 billion CFU QD), and Triphala Extract (250 mg QD). With this protocol, he continued to recover very slowly over the next 6 months and eventually achieved 60% wellness per his parents' ratings. However, Tyler's progress stalled in that range for 12 months with continued fatigue, gastrointestinal symptoms, mild tics, school avoidance, occasional rages, moderate anxiety, and continued (albeit less prominent) auditory hallucinations. Antibiotics and a third course of IVIG did not impact his symptoms. At that point Tyler's bacterial infection tests were negative and he complained of stomach pain. Antibiotics were discontinued. His treatment included nystatin (400,000–600,000 units 4 times daily), guanfacine (1 mg QD), a multivitamin/mineral, S-Acetyl Glutathione (200 mg QD), and CBT. Six months of CBT appeared to provide only minimal benefit for his residual symptoms. Though Tyler expressed appreciation for the emotional support in psychotherapy, he was unable to overcome his fear and avoidance of school.

After one year with the same physical and mental status, Tyler's parents and providers turned the focus of his treatment to gastrointestinal health as he complained of stomach pain, and constant presence of nausea and hunger at the same time. Laboratory testing of his stool revealed intestinal dysbiosis. Although earlier in his treatment stages Tyler's restricted eating and OCD would not allow for adjustments in his unhealthy eating habits, at his 60% recovered state, he was more agreeable to diet changes. When dietary and gastrointestinal health protocols were enacted in earnest, Tyler quickly experienced a boost in his recovery. For example, within two weeks of discontinuing dairy-containing foods, his “rages” and stomach aches improved. Within one month of eating an anti-inflammatory diet, including nutrient-dense shakes, his parents reported their son's wellness at 90%. Tyler reported more energy and resumed his recreational activities along with a new exercise program. Shortly thereafter Tyler returned to school. He continued in his improved state for three years without medications aside from probiotics. Tyler experienced a relatively mild “anxiety setback” immediately after receiving Pfizer vaccines for Sars CoV-19. At age 19, Tyler remains free of Sydenham's chorea, tics, PANS, OCD, or psychotic symptoms. He successfully completed high school, works full-time, and enjoys a social life.

## Discussion

The three young patients described in this paper experienced sudden-onset disabling neuropsychiatric symptoms and fully recovered following treatment of active infections along with relatively unconventional interventions (see Table 4 for complete list). During acute illness, all three patients met criteria for PANS and were housebound. One had lost developmental milestones including speech and ambulation. Another was diagnosed with Sydenham's chorea by a neurologist and suffered with auditory hallucinations and paranoia for 18 months. Two of the patients' improvements appeared to extend beyond their pre-morbid wellness and functioning, including Shelly's resolution of long-standing seizure activity and Mia's newfound agreeableness and flexibility.

**Table 4:** Treatments administered during period of recovery.

Intervention	Case study 1 “Mia”	Case study 2 “Shelly”	Case study 3 “Tyler”
IVIG			3 treatments
Guanfacine			✓
Ibuprofen			✓
Nystatin			✓
S-Acetyl-glutatione			✓
Modified citrus pectin		✓	
Digestive enzymes		✓	
Monolaurin		✓	
Resveratrol		✓	
Curcumin	✓		✓
Antibiotics	✓		✓
Herbal antimicrobials	✓	✓	
Cognitive-behavioral therapy	3 weeks	✓	✓
Magnesium	✓	✓	
Vitamin D		✓	✓
Mult-vitamin/multi-mineral	✓		✓
Nutrient-dense natural shakes		✓	✓
Fish oil/omega 3 fatty acids	✓	✓	✓
Probiotics	✓	✓	✓
Dietary modifications	✓	✓	✓

With regard to the origins of their neuropsychiatric conditions, uncontrolled case studies such as these are limited to observational commentary. It is known that two of three patients had a history of streptococcal infection but remained symptomatic long after resolution. All three patients had laboratory evidence of (undiagnosed and untreated) tick-borne diseases. Recoveries of their neuropsychiatric symptoms coincided with interventions aimed at treating infections, reducing inflammation, and improving gastrointestinal health. Other researchers have similarly reported relief of some psychiatric and neurological symptomatology through anti-inflammatory, infection, and/or immune-based interventions [e.g., (2, 28, 31)], as well as gastrointestinal treatments and dietary changes (32, 33). One patient improved during IVIG and antibiotic therapies, but his progress plateaued until treatment shifted exclusively to gastrointestinal care and anti-inflammatory eating. All three patients' protocols included antimicrobials, anti-inflammatory supplements, probiotics, and dietary changes. Two patients' protocols included antibiotics during the earlier phases of their illnesses. Two patients attempted treatment with psychotropic medications without benefit. Two patients partook in brief cognitive-behavioral psychotherapy, however, the timing of their improvements did not appear to coincide with psychotherapy. See Table 4 for complete list of treatments administered during patient recoveries.

Clarifying how or when severe psychiatric conditions are reversible, particularly through these less invasive, less costly, and curative interventions is paramount. Hope for this potential is supported by seminal research published in *Nature* from Kipnis's laboratory in which he and his colleagues discovered structural and functional connections between the peripheral immune and central nervous systems (CNS). This line of research uncovered that the brain is not as “immune-privileged” as previously thought (34), and unveils potential root causes with the authors stating that the link between the immune system and CNS “can develop pathological interactions that lead to neurological or psychiatric diseases” (35). Discovering sources of neuroinflammation leads to endless possibilities in finally determining origins and curative treatments for psychiatric and neurological conditions.

With increasing evidence connecting the immune system and brain, a paradigm shift in psychology, psychiatry, and neuropsychiatry is forthcoming. Until then, providers treating patients with sudden-onset, mysterious, and/or treatment-resistant psychiatric symptoms as reported in this paper, can consider infections, autoimmune, or other potential neuroinflammatory triggers in their differential diagnoses. Clinical interviews can establish risk factors. For example, the young patients in this case series were raised in Lyme endemic areas, had somatic complaints prior to their abrupt neuropsychiatric crises, and one had a known tick bite. It was prudent to include tick-borne disease testing in their evaluations.

The potential role of tick-borne diseases in triggering neuroimmune conditions is of particular concern for several reasons. First, the incidence of Lyme disease is estimated at 329,000 per year, with 5- to 9-year-old children being one of the most affected groups according to the CDC (36). Second, Lyme disease transmission can be missed as many people do not routinely check for tick attachment on their bodies. Even when tick bites are detected, some people do not seek medical advice or testing. Further, standard Lyme disease tests have suboptimal sensitivity, leaving even patients with clinical symptoms potentially untreated. Finally, anecdotal reports from clinicians suggest that Lyme disease can present differently in children than in adults, as one pediatrician told the author, “The only signs were anxiety and sleeplessness”. With the aforementioned obstacles, potential neuropsychiatric sequelae of untreated Lyme disease can be overlooked and misdiagnosed.

It behooves psychiatrists and pediatricians to consider untreated infections and misdirected immune responses in their differential diagnosis of patients exhibiting sudden onset neuropsychiatric symptoms, particularly when the symptoms are severe and disproportionate to patients’ life circumstances or unresponsive to standard treatments such as psychotherapy or psychotropic medications. Under these circumstances, clinicians can include a multi-systemic whole-person assessment in their diagnostic processes and familiarize themselves with scientific reviews of immunological causes of psychiatric disorders such as OCD (20).

For all psychiatric patients, when identified health conditions are understood and ameliorated to the best of clinicians’ abilities, patients can take full advantage of life with less suffering. This is true of children and adults with autism, who are, of course, at least equally vulnerable to misdirected immune responses affecting the brain. Two patients in this study had pre-morbid mild pervasive development disorders. Interestingly autism and PANS/PANDAS share disruption of the same area of the brain, the basal ganglia (37) and have other physiological commonalities [e.g., (38)]. A recent compelling post-mortem analysis revealed two thirds of autistic individuals' brains (*n* = 25) had lymphocyte cuffs and other telltale markers of brain inflammation which were markedly higher than age-matched neurotypical brain tissue [*n* = 30; (39)].

As different streams of research linking neuropsychiatric conditions with immunological factors have converged, conceptualizations of “psychiatric” disorders, mechanisms of action, and treatments are being reconsidered [e.g., (40–42)]. Anti-inflammatory medications and antibiotics have been shown to benefit some psychiatric patient groups, while certain effective psychotropic drugs inherently have antimicrobial and anti-inflammatory properties [e.g., (43–45)]. Taken together, these findings suggest that neuroinflammation, or at least systemic inflammation, is an appreciable component of some psychiatric conditions. In fact, Leboyer called for consideration of a new field, “immuno-psychiatry”, citing ample evidence of immune reactivity and multiple elevated markers of inflammation among severely affected patient groups, notably those suffering with suicidal behavior or bipolar disorder (2015).

This case series paper is limited in its generalizability due to its retrospective methodology, lack of control, confounding variables such as the possibility that patients “outgrew” their conditions, and single-case analysis. Furthermore, all three of the patients received concurrent treatments and it is impossible to determine which components of their treatment contributed to their recovery. Future single case studies could be conducted using a prospective and controlled methodology. With regard to group studies, future investigations could randomly assign treatment-resistant patients to “standard psychiatric and psychotherapy care” or “supplemental immuno-psychiatric care” groups for comparison of outcomes.

Treatment-resistant patients and their families live with extraordinary suffering, disability, and financial burden. Undetected and therefore untreated health conditions in children are of particular concern given increased vulnerability in youths as rates of autism, anxiety disorders, depression, and suicide among teenagers have risen. Appreciating connections between infections, neuroinflammation, and mental health disorders could prevent prolonged disability and reduce costs of psychiatric care.

## Data Availability

The original contributions presented in the study are included in the article/supplementary material, further inquiries can be directed to the corresponding author/s.
